# Supplementary material for: Plasma Vitamin B12 and Folate Alter the Association of Blood Lead and Cadmium and Total Urinary Arsenic Levels with Chronic Kidney Disease in a Taiwanese Population
Source: Nutrients. 2021 Oct 28;13(11):3841. doi: 10.3390/nu13113841 (PMC8625054; doi:10.3390/nu13113841)
Supplement: Supplementary file 1 [file nutrients-13-03841-s001.zip › nutrients-1406267 supplementary.pdf]

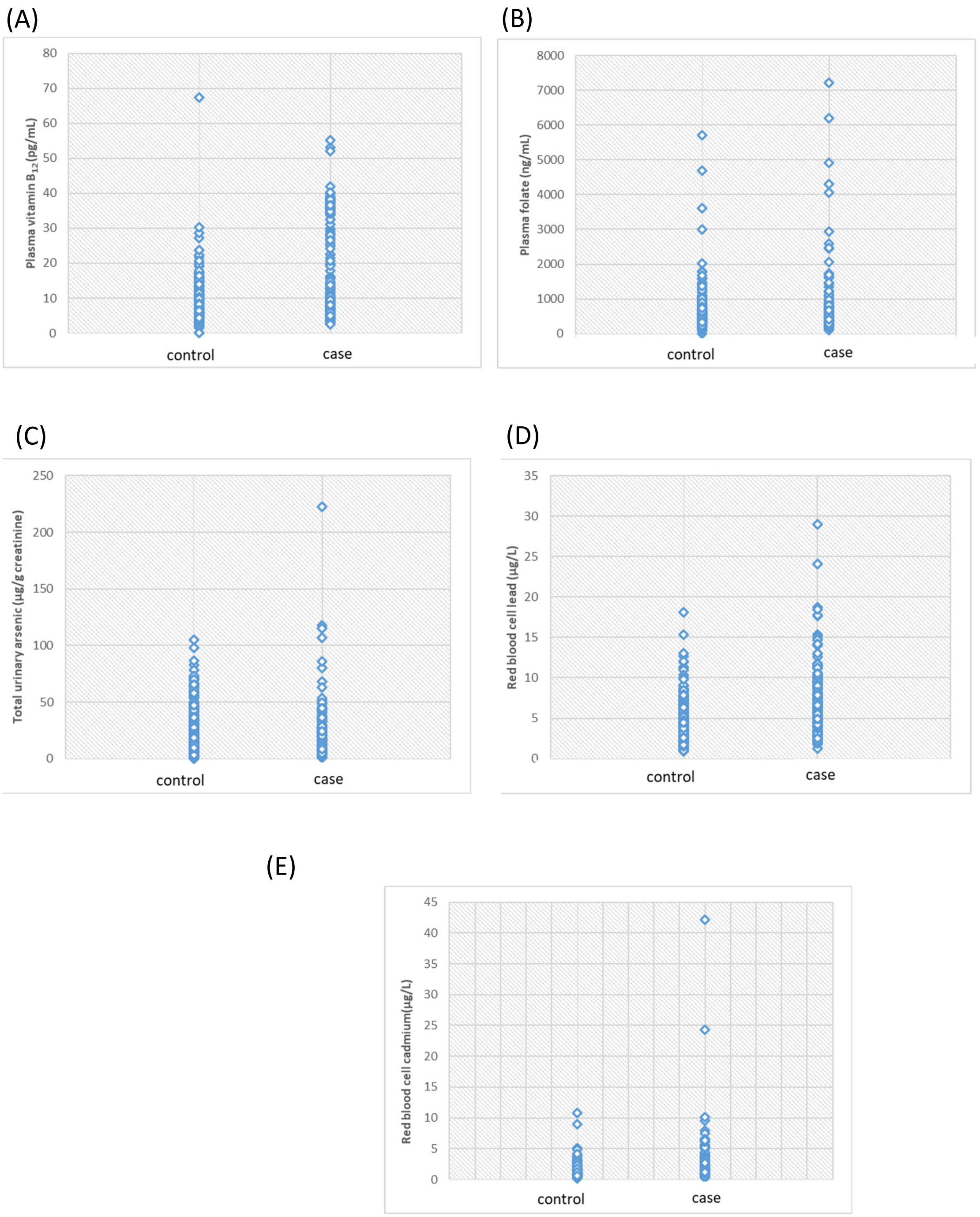

Supplementary Figure S1. The dot plots of measured variables by CKD status. (A) Plasma vitamin B12 (pg/mL) (B) Plasma folate (ng/mL) (C) Total urinary arsenic (µg/g creatinine) (D) Red blood cell lead (µg/L) (E) Red blood cell cadmium (µg/L).

Supplementary Tables

Supplementary Table S1. Validity and reliability of measurements used for determining urinary arsenic and plasma selenium, folate, and vitamin B_{12} as well as red blood cell lead and cadmium concentrations.

Metals or metalloids	Instrument	Detection limit (µg/L)	Recovery rate	Standard reference materials (SRM)	Coefficient of variance (CV)
Plasma folate and vitamin B_{12}	Radioassay kit (Bio-Rad, Richmond, CA, USA) and the 1470 Wizard series gamma counter	0.6 for plasma folate<br>75 pg/mL for vitamin B_{12}			Plasma folate: 9.3%<br>Plasma vitamin B_{12}: 6.8%
Red blood cell lead	Inductively coupled plasma mass spectrometry	0.32	100% ± 20%	SRM (Seronorm Trace Elements Whole Blood L-2 (Lot 1103129)) certificate value 310.0 µg/L (range: 186.0–434.0 µg/L); 329.0 ± 17.0 µg/L in our system	<5%
Red blood cell cadmium	Inductively coupled plasma mass spectrometry	0.07	100% ± 20%	SRM (Seronorm Trace Elements Whole Blood L-2 (Lot 1103129)) certificate value 5.8 µg/L (range: 5.4–6.2 µg/L); 6.1 ± 0.5 µg/L in our system	<5%
Arsenite (As^{III})	High-performance liquid chromatography–hydride generator–atomic absorption spectrometry	0.02	93.8%–102.2%	SRM [National Institute of Standards and Technology (NIST, Gaithersburg, MD, USA)] 2670 certificate value 480 ± 100 µg/L inorganic arsenic; in our system 507 ± 17 µg/L (n = 4)	<10%
Arsenate (As^{V})		0.10			
Monomethylarsonic acid (MMA^{V})		0.07			
Dimethylarsinic acid (DMA^{V})		0.06			
Urinary creatinine level	A colorimetric assay. The creatinine–picric acid complex formed from creatinine and picric acid was measured using a Roche Modular P800 instrument.				

Supplementary Table S2. Association of levels of total urinary arsenic and blood cadmium and lead with CKD stratified by B_{12} levels.

Plasma vitamin B_{12} level ≤ 7.76 pg/mL				Plasma vitamin B_{12} level > 7.76 pg/mL			
Variable	Case/Control	Age-sex adjusted ORs (95% CI)	Multivariate adjusted OR (95%CI)	Variable	Case/Control	Age-sex adjusted ORs (95% CI)	Multivariate adjusted OR (95%CI)
Total urinary arsenic (μg/g creatinine)				Total urinary arsenic (μg/g creatinine)			
≤11.91	17/75	1.00[§]	1.00[a]	≤12.07	53/76	1.00	1.00[a]
11.91–21.80	27/77	1.55 (0.77–3.09)	1.03 (0.41–2.58)	12.07–23.01	36/66	0.78 (0.45–1.33)	0.87 (0.39–1.96)
>21.80	54/76	3.13 (1.63–6.00)**	1.94 (0.81–4.69)	>23.01	33/68	0.68 (0.39–1.18)	0.65 (0.28–1.49)
Red blood cell lead (μg/L)				Red blood cell lead (μg/L)			
≤28.06	8/75	1.00[§]	1.00[b§]	≤27.76	12/70	1.00[§]	1.00[b§]
28.06–49.08	24/77	3.05(1.28–7.27)*	3.01 (0.99–9.19)	27.76–44.34	26/71	2.25(1.04–4.84)*	1.30 (0.44–3.88)
>49.08	66/76	8.75 (3.85–19.86)**	7.12 (2.44–20.75)**	>44.34	84/69	7.69 (3.79–15.61)**	3.74(1.34–10.46)**
Red blood cell cadmium (μg/L)				Red blood cell cadmium (μg/L)			
≤0.82	7/78	1.00[§]	1.00[b§]	≤0.74	10/70	1.00[§]	1.00[b§]
0.82–1.32	24/76	3.75 (1.50–9.34)**	3.13 (0.98–10.00)	0.74–1.26	27/72	2.98 (1.32–6.73)**	4.19 (1.25–14.00)*
>1.32	67/74	10.88 (4.58–25.83)**	9.28 (2.87–29.96)**	>1.26	85/68	10.47 (4.88–22.47)**	10.77 (3.19–36.33)**

[a]Adjusted for sex; age; educational level; alcohol, coffee, and tea consumption; analgesic use; diabetes; hypertension; and red blood cell lead and cadmium levels.
[b]Adjusted for sex; age; educational level; alcohol, coffee, and tea consumption; analgesic use; diabetes; hypertension; and levels of urinary creatinine, total urinary arsenic (μg/L), and red blood cell lead or cadmium.
*$P < 0.05$, **$P < 0.01$, and [§]$P < 0.05$ for the trend test.
